# A New High-Temperature Durable Absorber Material Solution through a Spinel-Type High Solar Absorptivity Coating on Ti_2_AlC MAX Phase Material

**DOI:** 10.1021/acsami.1c10585

**Published:** 2021-09-08

**Authors:** Wujun Wang, Fei Ye, Wangzhong Mu, Joydeep Dutta, Björn Laumert

**Affiliations:** † Department of Energy Technology, KTH Royal Institute of Technology, Stockholm 100 44, Sweden; ‡ Department of Applied Physics, KTH Royal Institute of Technology, Stockholm 114 19, Sweden; § Department of Material Science and Engineering, KTH Royal Institute of Technology, Stockholm 100 44, Sweden; ⊥ Center of Nanotechnology, King Abdulaziz University, Jeddah 21589, Saudi Arabia

**Keywords:** solar receiver, MAX phase, iron–cobalt–chromite spinel coating, spectral hemispherical absorptivity, concentrating solar power, thermal stability

## Abstract

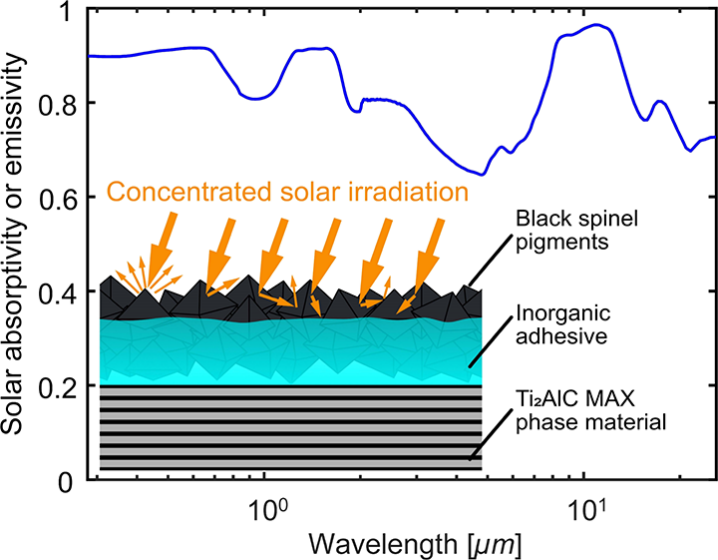

Enhancing the operating temperature of concentrating solar power systems is a promising way to obtain higher system efficiency and thus enhance their competitiveness. One major barrier is the unavailability of suitable solar absorber materials for operation at higher temperatures. In this work, we report on a new high-temperature absorber material by combining Ti_2_AlC MAX phase material and iron–cobalt–chromite spinel coating/paint. This durable material solution exhibits excellent performance, passing the thermal stability test in an open-air environment at a temperature of 1250 °C for 400 h and at 1300 °C for 200 h. The results show that the black spinel coating can offer a stable high solar absorptivity in the range of 0.877–0.894 throughout the 600 h test under high temperatures. These solar absorptivity values are even 1.6–3.3% higher than that for the sintered SiC ceramic that is a widely used solar absorber material. Divergence of solar absorptivity during these relatively long testing periods is less than 1.1%, indicating remarkable stability of the absorber material. Furthermore, considering the simple application process of the coating/painting utilizing a brush followed by curing at relatively low temperatures (room temperature, 95 and 260 °C in sequence), this absorber material shows the potential for large-scale, high-temperature solar thermal applications.

## 1 Introduction

Concentrating solar power (CSP) technology is one of the most promising pathways for a future fossil-fuel-free society because of the bountiful resource and its capability in providing electric baseload power upon integration with large-scale thermal storage facilities.^1,2^ Unlike photovoltaic technologies, the full spectrum of the solar radiation can be efficiently used in CSP technologies to convert into heat that can be used for industrial processes, water desalination, electricity generation via power cycles, and sustainable fuel production through thermochemical reactions.^3−5^ According to the second law of thermodynamics, increasing the working temperature of the power cycles is an important way forward for obtaining higher system efficiency and thus enhancing competitiveness of the CSP technologies in the future energy market. Indeed, this is considered as one of the main objectives identified to be achieved in the roadmap of the next-generation CSP technologies.^6^

Among the available power cycles, the Brayton cycle works at the highest temperature. Because of the high working temperature, it has its greatest potential in achieving high solar-to-electric net annual average conversion efficiency by combining with other power cycles, e.g., steam Rankine cycles and supercritical CO_2_ cycles.^7^ If the turbine inlet temperature could reach 1300 °C, 60% thermal efficiency is expected to be achieved by combined cycles.^7^ However, compared to the working temperature of the modern industrial gas turbines (>1450 °C),^8^ the maximum working temperature of the existing CSP Brayton systems are still considerably lower.^9^ A major technical barrier to achieve this arises from the solar air/gas receiver, which is required to sustain extreme working conditions for long periods of time. Highly concentrated solar fluxes (up to 1 MW/m^2^) leading to high temperatures and gradients, high solar flux variations causing thermal shocks due to the natural intermittent characteristic of the solar irradiation (mainly caused by local weather conditions, such as clouds, fog, etc.), high-temperature oxidation of materials, as well as problems arising from thermal stresses and fatigue. Especially in the absorber where the highest temperature is located, requirements for the high-temperature performances of the candidate materials are extremely challenging. Furthermore, the air/gas receivers can also be adapted as chemical reactors for solar thermochemical reactions, such as methane reforming reaction (>700 °C) and water/carbon dioxide splitting thermochemical reaction for hydrogen production (>1300 °C).^10−13^ Therefore, it is crucial to develop more durable, cost-efficient, and higher-performance material solutions for the future high-temperature air/gas receiver designs.

Conventionally, the receivers are either fabricated with refractory alloys or ceramics.^14^ With metallic materials, high-temperature oxidation is a major limiting factor, though the thermomechanical performances and thermal properties are reasonable. In practice, the maximum allowable working temperature of the refractory alloys in an open-air environment is generally below 1250 °C, which is much lower than the ceramic materials. Consequently, the outlet air temperatures of present metallic receivers are still located in the range of 800–900 °C for long duration usage.^9,15,16^ Compared to the metallic materials, ceramics offer much higher maximum working temperature (>1600 °C) due to their mechanical and chemical stabilities in high-temperature oxidizing environments. However, because of the brittle nature of ceramics, thermal shock resistance and fracture resistance are relatively low. Therefore, the risk of ceramic debris from absorber damage/failure is of significant relevance in determining the service life of high-speed gas turbines, which is currently considered to be a big challenge for the ceramic industry, although damage-tolerant design and manufacturing have been explored. Furthermore, the manufacturability of applicable ceramic materials is relatively poor, leading to higher manufacturing costs of large-scale receivers. Thus, in spite of the advances made with ceramic receivers reaching an outlet air temperature of 1100–1300 °C under a pressure of 3–20 bar,^17^ their large-scale application in commercial CSP systems is still limited. Thus, new material solutions that can combine the advantages of both metallic and ceramic materials to avoid either shortcomings will pave the way for wider application of CSP systems for fossil-free power generation.

One such attractive candidate is MAX phase materials, which are a class of ternary nanolayered early transition-metal carbides and nitrides. MAX phase materials are represented by the general formula M_*n*+1_AX_*n*_ (*n* = 1, 2, or 3), where M is an early transition metal, A is an A-group element, and X is either C (carbon) and/or N (nitrogen).^18^ The layered crystal structure enables the MAX phase materials to have a unique combination of both the characteristics of ceramics and metals. On the one hand, similar to their corresponding binary carbides and nitrides, the MAX phase materials are refractory materials (with good thermal stability up to 1600 °C in a vacuum)^19^ that are elastically stiff, resistant to oxidation, and have relatively low thermal expansion coefficients. On the other hand, MAX phase materials also exhibit metal-like properties, such as high thermal conductivity, excellent thermal shock resistance, and good machinability.^19,20^ All these characteristics can perfectly meet the material requirements of the modern high-temperature receiver designs. Thus far, more than 155 MAX phase materials, including solid-solution MAX phases, have been reported in the literature.^21^ Among them, Al-containing MAX phases, such as Ti_2_AlC, Cr_2_AlC, and Ti_3_AlC_2_, are considered to be the most suited for the high-temperature applications in an oxidizing environment because of their ability to form dense protective α-Al_2_O_3_ layers in an oxidizing environment at elevated temperatures because of their corundum structure.^22^ Especially for Ti_2_AlC, the thermal expansion coefficient is approximately 8.2 × 10^–6^ /°C,^23^ which is close to that of the α-Al_2_O_3_ protective layer, leading to minimal spallation failure of the protective layer caused by thermal stresses and thermal shocks.^24,25^ Furthermore, a significant self-healing phenomenon has also been observed on the α-Al_2_O_3_ protective layer at a temperature of 1200 °C, which is an added benefit.^22,26^ Thus, the combination of these two advantages allows the Ti_2_AlC material to be used over long working periods even at temperatures as high as 1400 °C in atmospheric conditions.^27^ Furthermore, Ti_2_AlC MAX phase materials are readily available commercially, rendering it easier to be used for wide-scale applications.^19^ As the Ti_2_AlC MAX phase material can be formed as coatings and porous foams.^28,29^ Therefore, the Ti_2_AlC MAX phase material can be applicable for almost all the existing solar receiver types, and recently, it has been introduced in the solar receiver design.^30^ However, considering that the protective Al_2_O_3_ layer plays a negative role in solar absorption,^31^ it limits the competitiveness of the Ti_2_AlC MAX phase material in the application of CSP solar receiver design for the future large-scale CSP applications.

In previous studies, surface texturing and refractory black coating/painting have been used as two main solutions for enhancing the solar absorptivity.^32−34^ Because of the self-healing phenomenon, the micro textured structure gets filled up with the growing Al_2_O_3_ layer quickly, thus reducing the probability of higher surface texture affecting solar absorptivity when utilizing Ti_2_AlC MAX phase materials.^26^ Thus, searching for high-temperature durable coatings/paintings with high solar absorptions for the Ti_2_AlC MAX phase material is of particular importance for future application of the MAX phase materials in the high-temperature solar receiver designs. To render new material solutions that are more competitive than traditional nickel-based superalloys, the working temperature of the coatings/paintings should be at least above 1250 °C.^35^ However, at these high temperatures, the choice of potential coating/painting with high solar absorption is very limited. Only a few ceramic and intermetallic materials might meet the requirements, such as black spinel pigments, silica carbide (SiC), and molybdenum disilicide (MoSi_2_).^36−39^ Furthermore, for the purpose of future large-scale application in CSP technologies, the coating/painting should also be low-cost and easily applicable on large-sized components. Therefore, a coating/painting solution that involves mixing black spinel pigment with inorganic adhesive is of great interest for improving the solar absorptivity performance of the Ti_2_AlC MAX phase material. However, as the MAX phase material is in the early stage of application, there is still no published literature about refractory-grade high solar absorptivity coatings with MAX phase materials. Thus, no operating experience or performance data can be directly used for solar receiver/reactor design.

In this work, we present a durable material solution, by combining commercial Ti_2_AlC MAX phase material and iron–cobalt–chromite spinel-based coating/painting, for future high-temperature CSP applications. The new durable material solution has successfully passed the thermal stability tests in an open-air environment at a temperature of 1250 °C for 400 h and 1300 °C for 200 h. The results show that the black spinel coating has excellent optical performance in a high-temperature environment. The solar absorptivity of the coating can reach the range of 0.877–0.894 with high stability during the total 600 h extremely high temperature tests carried out during this study. These solar absorptivity values are even higher than the SiC ceramic material that is widely used as the absorber material in the traditional high-temperature solar receiver/reactor designs. Considering the thermal expansion ratio of Ti_2_AlC MAX phase material (approximate 8.2 × 10^–6^ /°C) is close to some types of ceramics, more MAX phase materials and ceramic coatings could be explored for continuously improving the performances of future high-temperature CSP systems. Therefore, this work opens a door to a new solar absorber material family for high-temperature CSP applications.

## 2 Experimental Details

### 2.1 Material Selection and Coating Preparation

Commercial Ti_2_AlC MAX phase material (Maxthal 211), purchased from Kanthal AB, was used as the substrate material, whereas a commercial iron–cobalt–chromite spinel paint was selected as the coating material. Considering the future large-scale applications, brush painting and spray painting are two of the most preferred coating technologies. Hence, a commercial iron–cobalt–chromite spinel paint, Aremco’s HiE-Coat 840-MX (HiE), is used in this work because of its high-temperature stability, high emissivity, and possibility to be painted by a soft brush. Furthermore, the HiE paint costs 110 USD/Pint, and each pint can be used for coating a 20–25 m^2^ surface, thus fairly meeting the low-cost requirements for large-scale applications.

**Figure 1:**
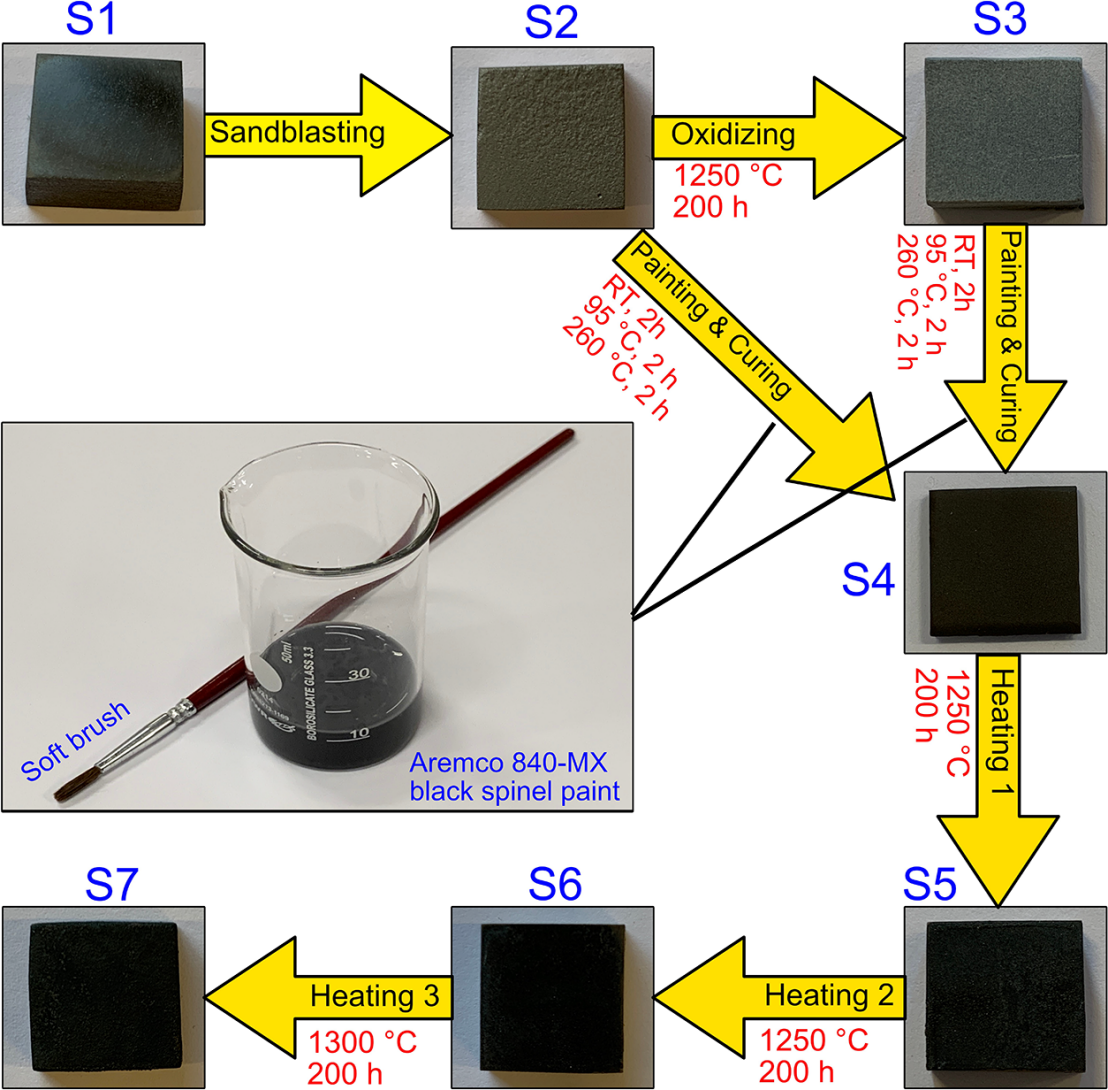
Schematic representation of the sample preparation process. S1: as-received samples without any surface treatment; S2: samples with a sandblasted surface; S3: sandblasted samples upon oxidization in air at 1250 °C for 200 h; S4: samples painted with Aremco’s HiE-Coat 840-MX, followed by curing at room temperature for 2 h, 95 °C for 2 h, and 260 °C for 2 h; S5: painted samples upon exposure to air at 1250 °C for 200 h; S6: painted samples upon exposure to air at 1250 °C for 400 h; S7: painted samples upon exposure to air at 1250 °C for 400 h and at 1300 °C for 200 h.

The sample preparation process is schematically represented in Figure 1. The Ti_2_AlC MAX phase substrate material was first cut into samples (S1) of size 15 × 15 mm and thickness of 5 mm. The surfaces of these samples were then sandblasted (S2) for obtaining better adhesion performance as suggested by the paint manufacturer as well as to obtain a diffused surface. After the sandblasting, some of the samples were oxidized in air at 1250 °C for 200 h (S3) in a high-temperature lab furnace (Thermconcept HTL 04/16) for two purposes. Optical performance of the oxidized surface (S3) was used as the reference for evaluating the enhancement in solar absorptivity of the coating. The oxidized samples (S3) were also painted with HiE coating, together with some sandblasted samples (S2) for comparing the thermal stability performances of the coating for two different surface treatments. Because the HiE black spinel paint is a commercial product that has been premixed with the liquid inorganic binder by the manufacturer, the coating process was directly achieved by a soft brush. After drying in air for 2 h at room temperature, the color uniformity was checked to ensure that the black coating has covered the whole sample front surface. All the selected samples were then subjected to curing at 95 °C for 2 h and subsequently at 260 °C for 2 h in the lab furnace as suggested by the manufacturer for optimal performance (all under atmospheric conditions). After curing, all the coated samples (S4) were heated in the high-temperature lab furnace for thermal stability tests. The complete testing procedure was chosen as 600 h in total: 400 h at 1250 °C and 200 h at 1300 °C. The temperature of the lab furnace is measured by a type B thermocouple (70%Pt/30%Rh–94%Pt/6%Rh). The final sample (S7) as well as the samples heated after 200 h (S5) and 400 h (S6) at 1250 °C were then studied in detail. To evaluate the photothermal performances of the HiE coating samples, we also measured the spectrum of hemispherical reflectance of a commercial sintered SiC ceramic (Hexology SA grade, Saint-Gobain) as the reference.

### 2.2 Material Characterizations and Optical Measurements

The crystal structure and the phase of coatings were examined by an Empyrean S2 diffractometer (PANalytical, The Netherlands) with Ni-filtered Cu Kα radiation (λ = 0.154 nm), and X-ray diffraction (XRD) data were collected in the range of 2θ ∼ 15–70°. The surface morphology of the samples were studied using scanning electron microscopy (SEM, GEMINI Ultra 55, Carl Zeiss, Oberkochen, Germany).

The spectrum of hemispherical reflectance from 0.25 to 2.5 μm was measured with a laboratory two-beam scan spectrophotometer (PerkinElmer Lambda 950) equipped with an Integrating Sphere of 150 mm diameter. Two light sources (halogen and deuterium lamp) and two detectors (photodiode and InGaAs) were used to cover the entire spectrum. All the measurements are referenced with a calibrated standard (Spectralon 99%). For the spectrum of hemispherical reflectance from 2.5 to 25 μm, a combination of a reflectometer (Surface Optics Corporation SOC-100 HDR) and a spectrophotometer (Nicolet FTIR 6700) was used. The SOC-100 is equipped with 2π imaging hemi ellipsoid (gold coated) to illuminate the sample from all directions using a 700 °C blackbody source. During the measurements, a gold-plated calibrated specular or diffuse reflectance standard is used as the reference.

### 2.3 Energy Analysis

Considering all the samples are opaque gray bodies, according to the Kirchhoff’s law, the spectral absorptivity/emissivity distributions of the investigated samples can be obtained by eq 1.
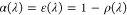
1where λ is the wavelength, α(λ) is the spectral absorptivity, ε(λ) is the spectral emissivity, and ρ(λ) is the spectral reflectivity. The normal solar absorptivity *α*_sol_ and the normal thermal emissivity ε(*T*) were calculated from the reflectance measurements using the following equations:
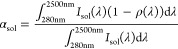
2
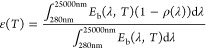
3where *T* is the surface temperature, *I*_sol_(λ) is the ASTM standard AM 1.5 direct normal terrestrial solar irradiance, and *E*_b_(*λ,T*) is the blackbody spectral emissive power, which follows Planck’s law, see eq 4.
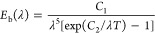
4where *C*_1_ = 3.742 × 10^8^ W μm^4^ m^–2^ and *C*_2_ = 1.439 × 10^4^ μm K are, respectively, Planck’s first and second radiation constants.

## 3 Results and Discussion

### 3.1 XRD Study

As seen in Figure 1, the surface color of the Ti_2_AlC MAX phase substrate material turns light gray upon exposure to air at 1250 °C for 200 h, suggesting the formation of the oxide layer. Furthermore, all the samples with the HiE coating have successfully passed the long period high temperature testing at 1250 °C for 400 h and 1300 °C for 200 h. No significant cracking, flaking, or surface color change could be observed upon prolonged exposure to atmospheric conditions at these high temperatures and thus the HiE coating can be painted directly on the sandblasted Ti_2_AlC MAX material surface without further surface oxidization treatment. This is important for large-scale applications, as Ti_2_AlC MAX material has excellent heat resistance and thus it would be difficult to oxidize its surface.

**Figure 2:**
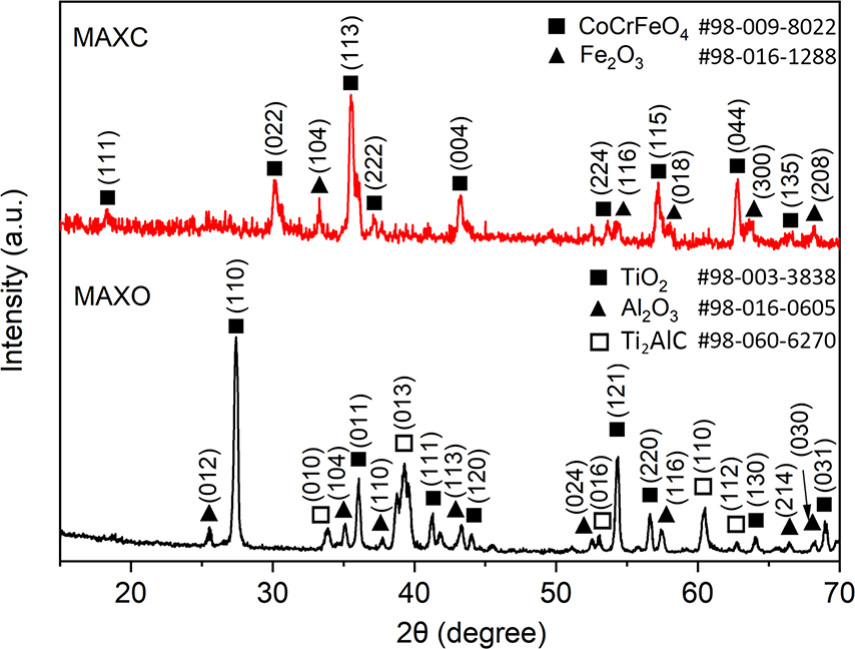
X-ray diffraction (XRD) patterns of substrate material sample S3 and coated sample S5 after being heated in air at 1250 °C for 200 h.

The XRD patterns of the surface-oxidized sample S3 and the coated sample that was exposed to air at 1250 °C for 200 h (S5) are shown in Figure 2. The surfaces of both the samples are composed of materials with stable mineral structures. In sample S3, the main phase structures of the oxidized layer formed are indexed as titania (TiO_2_) and alumina (Al_2_O_3_), in which TiO_2_ exhibits a higher diffraction intensity. Specifically, the most intense diffraction peaks at 2θ values of 27.4, 36.0, and 54.3° in sample S3 can be indexed to the (110), (011), and (121) planes, respectively, corresponding to the tetragonal rutile structure of TiO_2_ (ICDD card number 98-003-3838). Other peaks at 41.2, 44.0, 56.6, 64.1, and 69.0° are also identified and indexed respectively to (111), (120), (220), (130), and (031) diffraction planes of TiO_2_, respectively. Another oxide phase can be indexed as Al_2_O_3_ with diffraction peaks at 2θ of 25.5, 35.1, 37.7, 43.3, 52.5, 57.4, 66.4, and 68.1° matching well with ICDD card number 98-016-0605, corresponding to (012), (104), (110), (113), (024), (116), (214), and (030) crystal planes of the hexagonal corundum structure, respectively. Both TiO_2_ and Al_2_O_3_ are low solar absorptivity materials that limit the performance of the base material in harvesting solar energy, though the stable Al_2_O_3_ phase can offer good protection for the base material from further reactions with oxygen in air at extreme high temperatures. This is also a common drawback in all alumina-forming materials that limited their use in high-temperature CSP applications. Apart from the surface-oxidized layer, diffraction from the MAX phase substrate material, i.e., Ti_2_AlC, is also identified and indexed as a hexagonal structure (ICDD card number 98-060-6270). In coated sample S5, the major phase is identified as CoCrFeO_4_ with a cubic spinel structure. The most intense peak is at 2θ of 35.5°, which can be indexed as diffraction from (113) plane according to ICDD card number 98-009-8022. Other peaks at 2θ of 18.3, 30.2, 37.2, 43.2, 53.5, 57.2, 62.8, and 66.4° correspond to diffraction from (111), (022), (222), (004), (224), (115), (044), and (135) planes, respectively. Another phase in coating S5 with lower intensity than that of CoCrFeO_4_ is identified as Fe_2_O_3_ (ICDD card number 98-016-1288) of hexagonal structure. Diffraction peaks at 2θ of 33.3, 54.3, 57.8, 63.9, and 68.7° are indexed corresponding to the crystal lattices of (104), (116), (018), (300), and (208) planes, respectively.

### 3.2 Morphological Study

**Figure 3:**
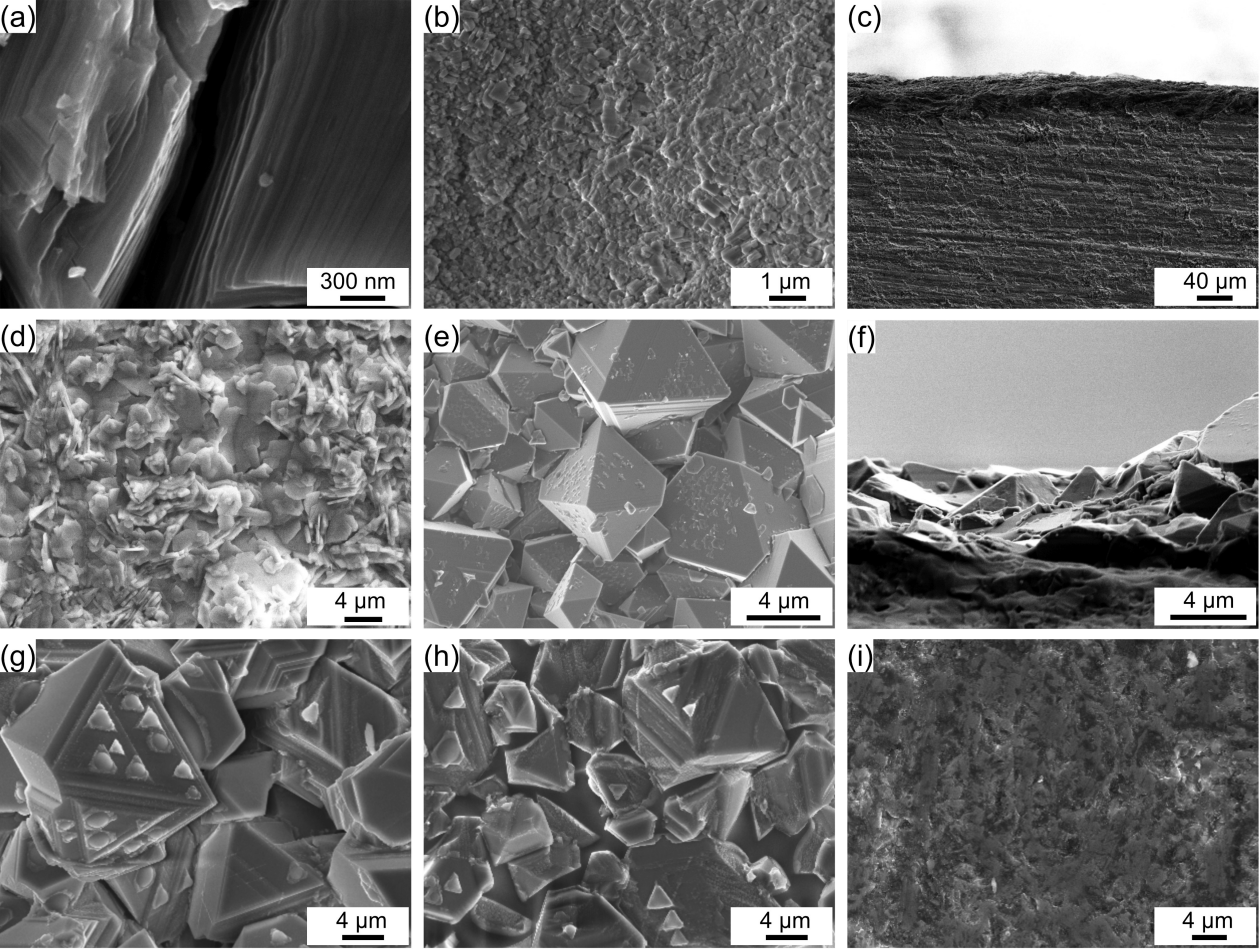
Scanning electron micrographs (SEM) of (a) Ti_2_AlC MAX phase substrate material (sample S1); (b) top view of the oxidized surface of Ti_2_AlC MAX phase material after exposure at 1250 °C in atmospheric environment for 200 h (sample S3); (c) cross section of the oxidized surface of Ti_2_AlC MAX phase material after exposing at 1250 °C in open-air environment for 200 h (sample S3); (d) top view of the HiE coating after curing but before high-temperature heat treatment (sample S5); (e) top view of the HiE coating after 200 h heat treatment at 1250 °C in open-air environment (sample S5); (f) cross section of the HiE coating after 200 h heat treatment at 1250 °C in open-air environment (sample S5); (g) top view of the HiE coating after 400 h heat treatment at 1250 °C in open-air environment (sample S6); (h) top view of the HiE coating after 400 h heat treatment at 1250 °C and 200 h at 1300 °C in open-air environment (sample S7); (i) top view of the commercial sintered SiC ceramic (Hexology SA grade, Saint-Gobain).

SEM images of the as-received Ti_2_AlC MAX phase material (S1), oxidized surface (S3), the coated surface after curing (S4), after 200 h of heat treatment at 1250 °C (S5), after 400 h of heat treatment at 1250 °C (S6), after 400 h of heat treatment at 1250 °C, and 200 h at 1300 °C, as well as commercial sintered SiC ceramic (Hexology SA grade, Saint-Gobain) are shown in Figure 3. Thicknesses of the coatings are in the range of 15–30 μm as shown in the side-view SEM images. The Ti_2_AlC MAX phase material (S1) exhibits a clear 2D-layered structure of micrometer-sized planar dimensions and nanosized dimensions in thickness, as can be observed in Figure 3a–c showing the top view and the cross-sectional view of the oxidized surface (S3) of the Ti_2_AlC MAX phase material after exposure at 1250 °C to atmospheric conditions for 200 h. An approximate 40 μm dense and continuous oxidation layer can be observed without any visible defects, providing efficient protection from further oxidation to the Ti_2_AlC MAX phase material. From the XRD studies, the main compositions of this oxidation layer are found to be TiO_2_ and Al_2_O_3_, which are not conducive to enhancing the solar absorptivity. Figure 3d shows the top view of the HiE coated sample after curing but prior to the high-temperature heat treatment (S5), where the micrometer-sized flaked pigments are clearly observed to be glued by the inorganic adhesive. After 200 h heat treatment at 1250 °C, CoCrFeO_4_ with a cubic spinel structure is formed as shown in Figure 3e and the microstructure increases to dimensions of 4–8 μm for one edge of the spinel. The side view of S5 coating in Figure 3f shows its spinel microstructure. It is clear that the pyramid-like surface microstructure on the coated surface is composed of these CoCrFeO_4_ spinel particles. With increased heating time, the average size of these CoCrFeO_4_ spinel particles increase, whereas the pyramid-like surface microstructure does no change, as shown in Figure 3g. However, after exposing the coated sample S6 at 1300 °C in an open-air environment for extra 200 h, a small number of secondary crystals are found to form on the bigger crystals, indicating the high thermal stability of the coating.

**Figure 4:**
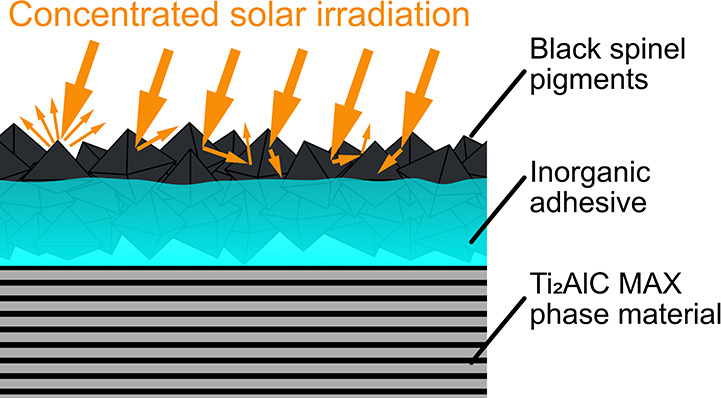
Schematic representation of the absorption, reflection, and scattering of concentrated solar irradiation on the HiE coating surface.

In previous studies, a similar pyramid-like surface microstructure has been shown to be an effective way to enhance solar absorptivity.^40^ This enhancement in absorptivity arises from the “cavity effect” formed between neighboring peaks that allows solar irradiation to be absorbed efficiently because of multiple reflections in these cavities. Figure 4 schematically represents the absorption, reflection, and scattering of the concentrated solar irradiation on the HiE coating surface. As shown in Figure 3e, f, the size of the iron–cobalt–chromite spinel crystal is generally in the range of 2–10 μm. Considering that around 95% of terrestrial solar radiation is located in the spectrum with wavelength less than 2 μm, and 72% is located in the spectrum with wavelength less than 1 μm, the most concentrated solar irradiation will be multireflected and absorbed by the faces of the spinel crystal due to the “cavity effect”.^41^ With a small portion (<5%) of the solar spectra having wavelengths longer than the size of the micro pyramids, Mie scattering would have a negligible impact on the total solar absorptivity.

### 3.3 Spectral Optical Properties

**Figure 5:**
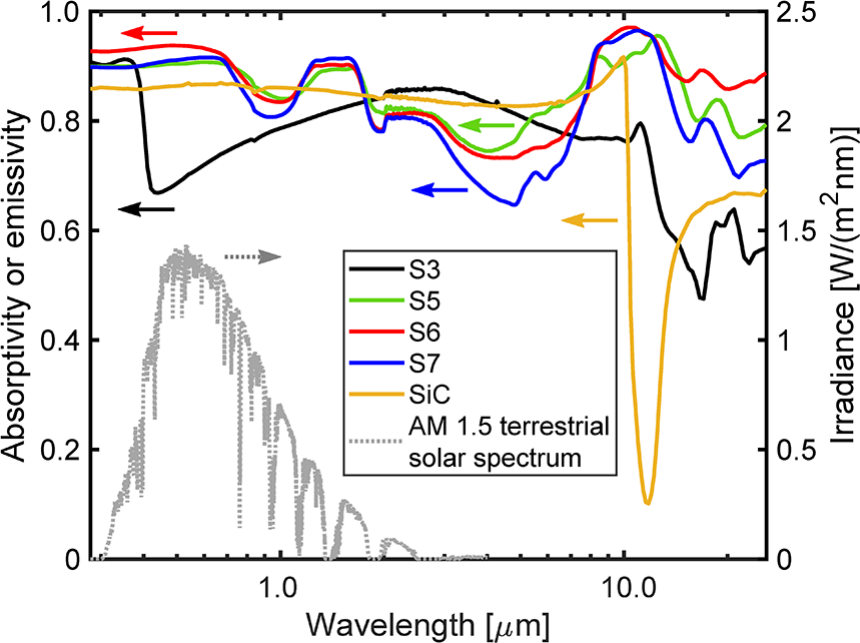
Spectral absorptivity results for the investigated samples. S3: sandblasted sample upon oxidation in air at 1250 °C for 200 h; S5: painted sample upon exposure to air at 1250 °C for 200 h; S6: painted sample upon exposure to air at 1250 °C for 400 h; S7: painted sample upon exposure to air at 250 °C for 400 h and at 1300 °C for 200 h; SiC: commercial sintered SiC ceramic (Hexology SA grade, Saint-Gobain).

The spectral absorptivity results of the investigated samples and the Hexology SA grade SiC ceramic are shown in Figure 5 together with the AM 1.5 terrestrial solar spectrum. In the wavelength range where the most solar radiation is located, the absorptivity of the HiE coating samples (S5, S6, and S7) is generally above 0.8. In the bands 0.28–0.72 μm and 1.25–1.65 μm, the absorptivity reaches 0.9 or even higher. Upon oxidation (sample S3), the absorptivity of the solar spectrum is significantly reduced compared to the coated samples. Especially in the visible light spectrum, 0.42–0.72 μm, where the energy-density peak of the solar irradiation is located, the absorptivity is lower than 0.75. Considering that the oxidized Ti_2_AlC MAX phase material consists mainly of Al_2_O_3_ and TiO_2_, the absorptivity of the oxidized surface could be even lower after being exposed in air for a longer time because of the growth of the oxide phases. In the wavelength range of 1.8–5.3 μm, the oxidized surface in sample S3 can show significantly higher absorptivity than that of the coated samples. Besides, the absorptivity decreases significantly with the exposure time at high temperature in this wavelength range. However, considering the energy density of the solar irradiance in this band is already very weak, the reduction in absorptivity in this wavelength range will not affect solar absorption significantly. It is worth noting that at high temperatures (800–1300 °C), the thermal radiation becomes very strong as explained by the Wien’s displacement law in this wavelength range.^42^ The thermal emissivity in this wavelength range could influence the radiative heat transfer between the absorber surface and the surrounding environment. However, considering that modern high-temperature receivers/reactors are usually designed with very high concentration optical concentrators and cavity-shaped absorbers, the influence of the thermal emissivity of the absorber material to the receiver/reactor efficiency is relatively low, compared to the solar absorptivity.^41,43^ Therefore, the absorptivity data in this wavelength range is more useful for the internal radiation heat-transfer design as the radiative heat transfer is non-negligible when the temperature exceeds 500 °C, and the high thermal emissivity surface can enhance the heat transfer on the heat sink side.^42^ For pressurized indirectly irradiated air receivers,^44−46^ the HiE coating can be applied only on the outer surface of the absorber, leaving the inner surface directly exposed to the hot gas as the oxidized surface can offer higher thermal emissivity.

Comparing the spectral absorptivity results of the HiE-coated samples to the Hexology SA grade SiC ceramic, the coated samples are able to offer higher absorptivity in the wavelength range of 0.28–1.8 μm, where most of the solar irradiance is located, except in the narrow band of 0.76–1.16 μm. In the wavelength range of 1.8–7.0 μm where the major thermal emissive energy of a hot surface (800–1300 °C) is located, the emissivity of the SiC ceramic is significantly higher than the HiE-coated samples. Therefore, the HiE coating can offer a better photothermal performance for solar absorber applications than the SiC ceramic: higher solar absorptivity and lower thermal emissivity.

**Figure 6:**
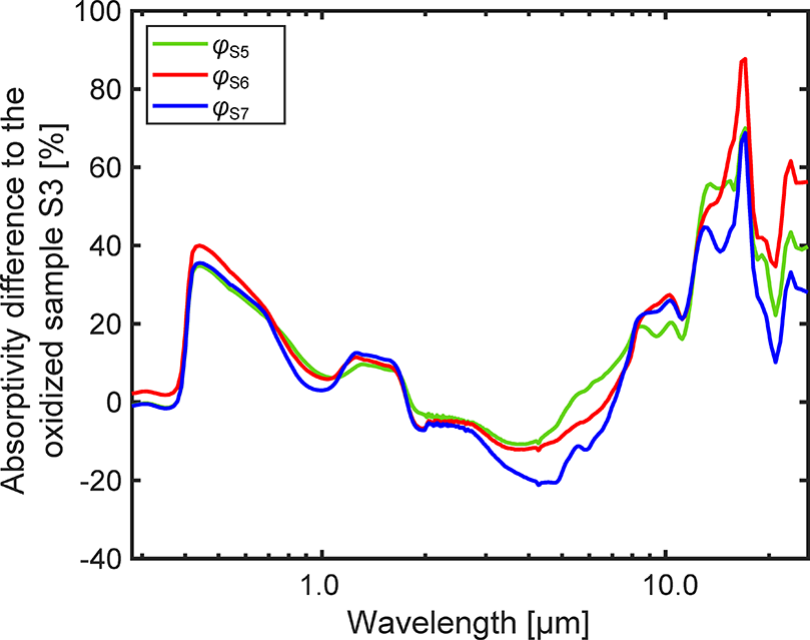
Absorptivity difference between the three coated samples (S5, S6, and S7) to the oxidized sample S3. φ_S5_: absorptivity difference between the painted sample S5 (upon exposure to air at 1250 °C for 200 h) and the oxidized sample S3 (sandblasted sample upon oxidation in air at 1250 °C for 200 h); φ_S6_: absorptivity difference between the painted sample S6 (upon exposure to air at 1250 °C for 400 h) and the oxidized sample S3 (sandblasted sample upon oxidation in air at 1250 °C for 200 h); φ_S7_: absorptivity difference between the painted sample S7 (upon exposure to air at 1250 °C for 400 h and at 1300 °C for 200 h) and the oxidized sample S3 (sandblasted sample upon oxidation in air at 1250 °C for 200 h).

To further investigate the absorptivity enhancement upon the application of the HiE coating on the Ti_2_AlC MAX phase material, we introduced a new parameter: φ (absorptivity difference to the oxidized sample S3) as given in eq 5.
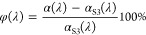
5As shown in Figure 6, the evolution of φ_S5_, φ_S6_, and φ_S7_ shows similar trends with respect to the irradiation wavelength. The HiE coating has a significant positive effect in enhancing the absorptivity in the wavelength range of 0.38–1.76 μm, where most of the solar irradiance is located. Especially in the visible light spectrum 0.42–0.72 μm, an average enhancement of more than 27% is reached, with peaks above 35%. Another significant enhancement peak is located in the wavelength range longer than 7 μm. However, for high-temperature applications, the thermal radiation energy portion in this wavelength range is pretty small and hence can be neglected. Consequently, for the Ti_2_AlC MAX phase material, the photothermal performance in the solar spectrum is thus enhanced upon the usage of the HiE coating on its surface.

### 3.4 Thermal Stability Analysis

The temperature distribution on the absorber surface is a combination of the absorbed concentrated solar irradiation distribution and the heat transfer design on the heat sink (working fluid) side. For a given receiver design, the heat transfer coefficient distribution on the side of the heat sink is already fixed. Any change in the absorptivity of the absorber surface would lead to unexpected issues, including efficiency reduction, increased thermal stress, and receiver failures due to local overheating. Therefore, the stability of the solar absorptivity of the absorber surface is extremely important in CSP applications.

**Figure 7:**
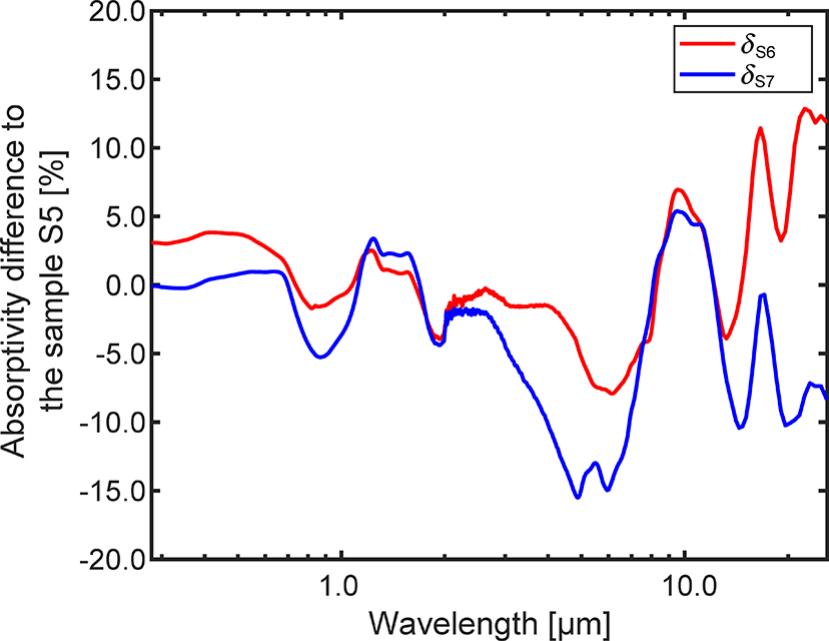
Absorptivity difference between the coated samples S6 and S7 to the coated sample S5. δ_S6_: absorptivity difference between the painted sample S6 (upon exposure to air at 1250 °C for 400 h) and the painted sample S5 (upon exposure to air at 1250 °C for 200 h); δ_S7_: absorptivity difference between the painted sample S7 (upon exposure to air at 1250 °C for 400 h and at 1300 °C for 200 h) and the painted sample S5 (upon exposure to air at 1250 °C for 200 h).

The coating surfaces of the samples were carefully checked after every thermal test under different temperature levels and periods. No significant cracking, flaking, or surface color change could be observed in samples S5, S6, and S7. Therefore, more detailed quantitative studies have been conducted by introducing an extra parameter in this study, δ (absorptivity difference from sample S5), as given in eq 6.
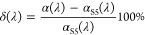
6Figure 7 shows that the stability of the spectral absorptivity is excellent in the wavelength range of 0.28–3 μm where the major energy of the solar irradiation located. The changes in the absorptivity are within ±5% in this wavelength range after heating the samples at 1250 °C for 400 h (S6) and even for an extra 200 h at 1300 °C. The small fluctuation in the stability of the absorptivity in the solar spectrum is negligible. For real applications, this small fluctuation can be easily managed by introducing suitable design margins for the absorber temperature peak. For the wavelength range of >3 μm, even though the contribution from the solar radiation is not significant, results show that the absorptivity changes are still within a relatively small range (approximately ±15%). Hence, it can be concluded that the thermal stability of the iron–cobalt–chromite spinel black coating on the Ti_2_AlC MAX phase material is excellent.

### 3.5 Solar Absorptivity and Thermal Emissivity

**Figure 8:**
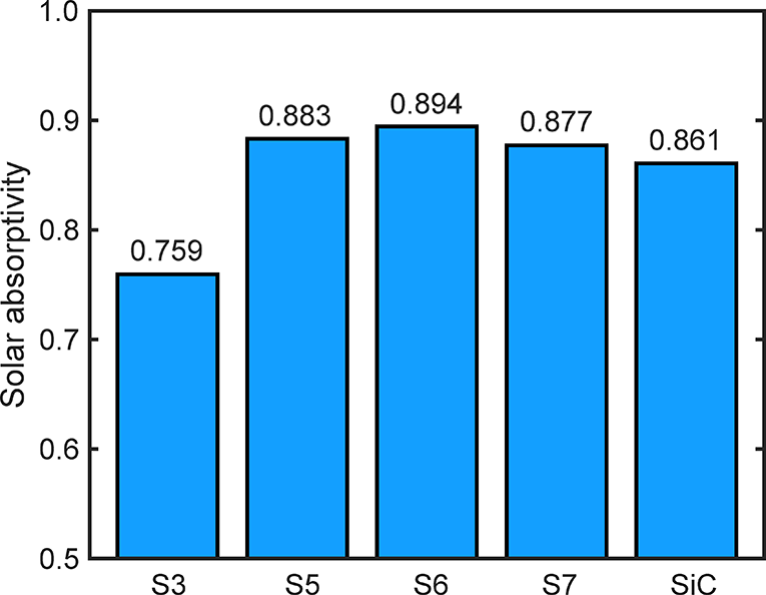
Solar absorptivity results for the investigated samples. S3: sandblasted sample upon oxidation in air at 1250 °C for 200 h; S5: painted sample upon exposure to air at 1250 °C for 200 h; S6: painted sample upon exposure to air at 1250 °C for 400 h; S7: painted sample upon exposure to air at 250 °C for 400 h and at 1300 °C for 200 h; SiC: commercial sintered SiC ceramic (Hexology SA grade, Saint-Gobain).

Normalized solar absorptivity and thermal emissivity was calculated on the basis of the spectral absorptivity, AM 1.5 terrestrial solar spectrum, and blackbody thermal emission according to eqs 2 and 3. The normalized solar absorptivity results of the investigated samples (S3, S5, S6, and S7) are shown in Figure 8. The solar absorptivity values of the coated samples are located in a very narrow range of 0.877–0.894, while the oxidized surface can only reach a value of 0.759. These results thus confirm the three most important findings that make the new material solution highlighted in this work as a potential candidate in high-temperature solar receiver/reactor applications. First, the HiE coating can offer excellent solar absorptivity that is already 1.6–3.3% higher than the most widely used ceramic absorber material (SiC). Second, the stability of black spinel HiE coating is excellent since even after a total of 600 h exposure in the high-temperature open-air environment (400 h at 1250 °C and 200 h at 1300 °C), the solar absorptivity change is less than 1.1%. In real world applications, this small solar absorptivity change is negligible. Finally, though the Ti_2_AlC MAX phase material has good performances in high-temperature oxidizing environment, the formed protective oxidizing layer has a poor performance in absorbing solar energy efficiently, which was circumvented by applying the black spinel HiE coating, increasing the solar absorptivity by 12%.

**Figure 9:**
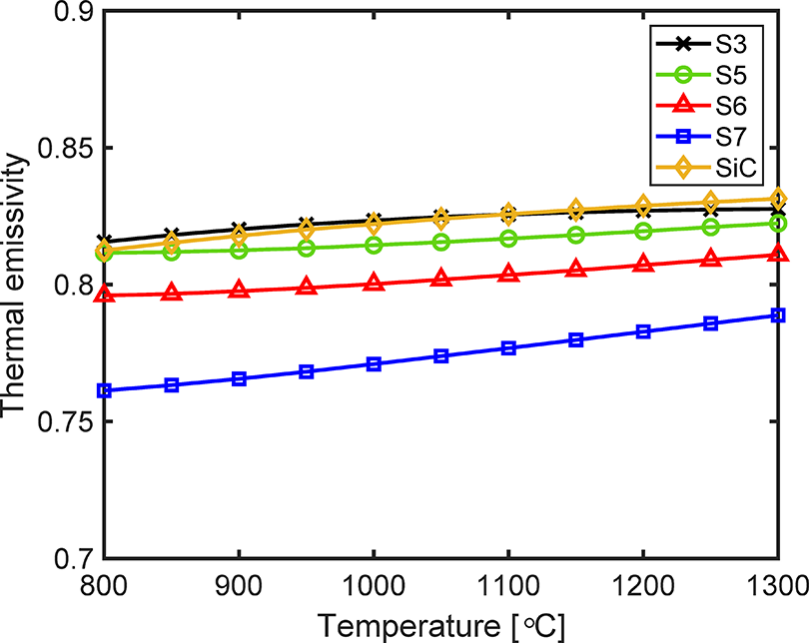
Thermal emissivity results for the investigated samples. S3: sandblasted sample upon oxidation in air at 1250 °C for 200 h; S5: painted sample upon exposure to air at 1250 °C for 200 h; S6: painted sample upon exposure to air at 1250 °C for 400 h; S7: painted sample upon exposure to air at 250 °C for 400 h and at 1300 °C for 200 h; SiC: commercial sintered SiC ceramic (Hexology SA grade, Saint-Gobain).

Although the thermal emissivity is not as important as the solar absorptivity in determining the efficiency of high-temperature receiver/reactor designs due to the high concentration ratios, it still plays an important role in the heat transfer design in the heat sink side. Therefore, the emissivity results of the investigated samples in the temperature range of 800–1300 °C were calculated as shown in Figure 9. Unlike the solar absorptivity, the thermal emissivity is a temperature-dependent parameter due to temperature dependence of the thermal emission spectrum. In general, all four selected samples share the same monotonically increasing trend with temperature. Compared to the coated samples, the oxidized surface and the SiC ceramic offer higher thermal emissivity, which might lead to an increase in radiative heat losses. For the coated samples, with the increase in atmospheric exposure time at higher temperatures, the thermal emissivity decreases slightly. Thus, to maximize the radiation heat transfer on the heat sink side, oxidation of the bare surface in air is possibly the best choice.

## 4 Conclusions

In conclusion, we have developed a new durable material solution for future high-temperature CSP applications by combining Ti_2_AlC MAX phase material and iron–cobalt–chromite spinel coating/paint. The durable material solution has successfully passed the thermal stability testing in an open-air environment at a temperature of 1250 °C for 400 h and 1300 °C for 200 h. Because of the high spectral absorptivity offered by the iron–cobalt–chromite spinel material itself and its pyramid-like surface microstructure, the black spinel coating can offer a stable high solar absorptivity in the range of 0.877–0.894 as observed during the total of 600 h high-temperature testing. These solar absorptivity values are even 1.6–3.3% higher than the sintered SiC ceramic (Hexology SA grade, Saint-Gobain) that is widely used as the solar absorber material in the high-temperature solar receiver/reactor designs. The solar absorptivity change during the long testing period is less than 1.1%. Compared to the oxidized surface of the Ti_2_AlC MAX phase material, the black spinel coating enhances the solar absorptivity by 12%. Furthermore, taking into account the simple coating/painting process by brushing and curing at relatively low temperatures (room temperature, 95 and 260 °C), this new absorber material solution can be easily up scaled for large-scale high-temperature CSP applications. Considering the thermal expansion ratio of Ti_2_AlC MAX phase material (approximate 8.2 × 10^–6^ /°C) is close to some other types of ceramics, we will further explore MAX phase materials and ceramic coatings for continuously improving the performances of future high-temperature CSP systems.
